# Copolymer Micelles: A Focus on Recent Advances for Stimulus-Responsive Delivery of Proteins and Peptides

**DOI:** 10.3390/pharmaceutics15102481

**Published:** 2023-10-17

**Authors:** Thomas Trimaille, Bernard Verrier

**Affiliations:** 1 Ingénierie des Matériaux Polymères, Univ Lyon, CNRS, Université Claude Bernard Lyon 1, INSA Lyon, Université Jean Monnet, UMR 5223, CEDEX, 69622 Villeurbanne, France; 2 Laboratoire de Biologie Tissulaire et d’Ingénierie Thérapeutique, Univ Lyon, CNRS, Université Claude Bernard Lyon 1, UMR 5305, 7 Passage du Vercors, CEDEX 07, 69367 Lyon, France; bernard.verrier@ibcp.fr

**Keywords:** copolymer micelles, self-assembly, micelles, proteins/peptides, stimuli-responsive

## Abstract

Historically used for the delivery of hydrophobic drugs through core encapsulation, amphiphilic copolymer micelles have also more recently appeared as potent nano-systems to deliver protein and peptide therapeutics. In addition to ease and reproducibility of preparation, micelles are chemically versatile as hydrophobic/hydrophilic segments can be tuned to afford protein immobilization through different approaches, including non-covalent interactions (e.g., electrostatic, hydrophobic) and covalent conjugation, while generally maintaining protein biological activity. Similar to many other drugs, protein/peptide delivery is increasingly focused on stimuli-responsive nano-systems able to afford triggered and controlled release in time and space, thereby improving therapeutic efficacy and limiting side effects. This short review discusses advances in the design of such micelles over the past decade, with an emphasis on stimuli-responsive properties for optimized protein/peptide delivery.

## 1. Introduction

Proteins and peptides constitute a major class of therapeutics, representing one of the fastest-growing sectors in the pharmaceutical industry, with hundreds of protein-based drugs approved and in clinical trials [1,2]. Being molecules typically produced by the body to ensure normal function, they have an inherent “non-toxic” profile. However, their biological function is closely related to their tertiary structure, which is fragile and easily altered. In addition, they have numerous charged groups and (in most cases) high hydrophilicity that can disfavor cell membrane permeability for reaching their target. Finally, they have a short half-life in vivo, impeding prolonged therapeutic effects [3].

Many nano-based delivery systems have been developed with more or less success to overcome these problems [4], including polymer nanoparticles [5,6,7,8] or micelles [9,10,11,12,13], polymersomes [14,15], lipid-based nanoparticles [16,17,18], hybrid lipid/polymer particles [19], and inorganic particles [20]. For example, particles from FDA-approved aliphatic polyesters such as polylactide (PLA) and poly(lactide-co-glycolide) (PLGA) have been extensively used for protein delivery, with several formulations marketed [4]. However, encapsulation of such hydrophilic macromolecules requires a process of double emulsion (water/oil/water) for particle elaboration, during which the integrity of the protein is partly altered as described more than 20 years ago [21]. In addition, the protein loading is rather low in this process. Lipid-based nanoparticles (liposomes or lipid nanoparticles) have also been described historically [16,22]. While the chemical engineering of these carriers has greatly evolved, as shown by the success of mRNA vaccines in the context of COVID-19, issues remain regarding storage stability and the achievable low protein loadings [23].

Among the abovementioned systems, micelle structures, arising from the self-assembly of amphiphilic copolymers, have gained considerable interest for protein/peptide delivery over the past decades [8,24,25]. They indeed present advantageous features including low size (<100 nm), stealth character (hydrophilic corona), ease of preparation, good reproducibility, and particularly high chemical versatility, as hydrophobic/hydrophilic segments can be tuned to exhibit suitable core design, surface charge, and functionalities for conjugation of biomolecules (drug, targeting moieties). Notably, such a tunability in copolymer design has largely benefited from the ground-breaking progress in controlled polymerization techniques (i.e., atom transfer radical polymerization (ATRP), reversible addition fragmentation transfer (RAFT), and nitroxide mediated polymerization (NMP)) since the end of 1990s, making possible many block copolymer natures which were until then unachievable, and with a high degree of control over chain, length, composition, and functionality [26,27]. Micelles have been traditionally regarded as choice carriers for hydrophobic drugs, such as many anticancer drugs, through physical encapsulation in the micelle core [28,29]. However, innovative strategies have emerged to load hydrophilic compounds such as proteins/peptides, either covalently or non-covalently (electrostatic, hydrophobic interactions, H-bonds), taking advantage of their versatility in polymer design [8,25]. Specifically, micelles here present a strong asset by being able to combine both the hydrophobic drug and protein, having complementary therapeutic effects to synergistically enhance treatment efficiency, particularly in the context of cancer [30].

The major challenge of drug delivery is promoting precise, selective delivery to the target, to avoid undesired side effects and maximize the therapeutic effect at minimal doses. In this context, besides introducing on carriers specific ligands of cell receptors (“active targeting” strategy) [31,32], the approach based on stimuli-responsive (“smart”) delivery systems to trigger drug release has currently been receiving tremendous attention since 2013 [33]. Proteins and peptides, as a highly attractive class of drugs, are no exception to this trend. Precisely, copolymer micelles are relevant for the purpose of smart, “on demand” delivery because their chemical flexibility allows them to easily build up and tune copolymer architectures with adapted stimulus-responsive behavior [34], which motivated the present review as none so far dealt with this topic. Undoubtedly, pH has been, to date, the most investigated internal stimulus to trigger drug delivery, not only for anticancer therapy (as there is an acidic pH in tumor environments) [35,36], but also in other pathological contexts, such as ischemia [37], bacterial biofilms [38], and inflammation [39], where pH is lower than that observed in normal tissues. Furthermore, as pH decreases following endocytosis in the endosomal compartments, this stimulus can be used to improve endosomal escape (e.g., through the proton sponge effect) and the protein drug is released intracellularly to reach its target [40]. This intracellular delivery is particularly important in the case of antigen proteins (in antigen-presenting cells, APCs) for eliciting a cellular immune response, which is highly desired in vaccine development against infectious diseases and cancer [41]. Other stimuli such as temperature [42], redox [43], and more recently enzymes [44] have also appeared as interesting stimuli to trigger drug release, taking advantage of hyperthermia, a reductive intracellular environment, and upregulation of enzymes in pathological sites, respectively. In this review, we aim at discussing the advances in stimulus-responsive micelles for protein/peptide delivery over the past decade. It should be mentioned that the delivery of nucleic acids with smart micelles, recently widely investigated, will deserve another review.

## 2. Non-Covalent Association to Micelles

Proteins can be loaded on/in micelles through a non-covalent approach. The general advantage is a simple and benign process as compared to a covalent strategy, for which possibly deleterious reaction conditions (e.g., toxic coupling agents, irradiation) can lead to partial protein denaturation or undesired side reactions. Nevertheless, it is well known that even passive (i.e., non-covalent) immobilization of proteins can have a dramatic effect on biological activity as the tertiary structure can be altered. Still, proteins released from micelles present a generally well-conserved biological activity. Non-covalent association generally involves hydrogen bonding, a hydrophobic effect, and electrostatic interactions. The latter has been particularly used to develop pH-triggered release systems, by controlling the attraction/repulsion electrostatic forces between the protein and dedicated polyelectrolyte as a function of pH. Temperature-sensitive as well as multiple stimuli-responsive micelles have also been reported.

### 2.1. pH-Repsonsive Micelles

**Figure 1:**
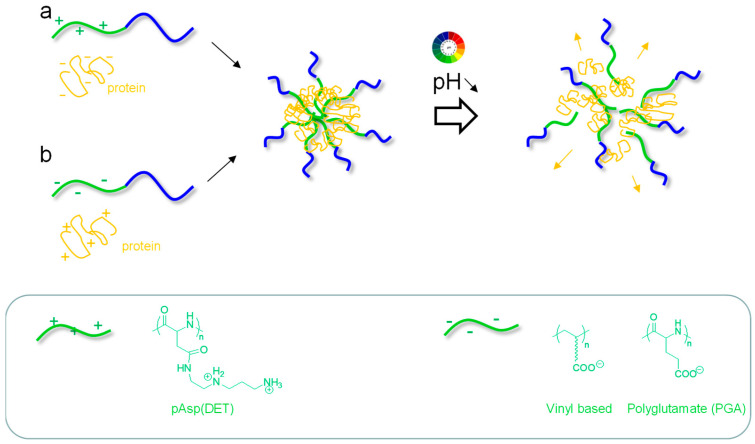
pH-responsive micelles based on polyion complex (PIC) micelles (the box shows the typical cationic and anionic segments used).

Polyion complex (PIC) micelles are attractive systems for pH-triggered release properties. Historically developed by Kataoka’s team [45,46], they are formed through electrostatic interactions when a diblock copolymer containing a hydrophilic neutral block (e.g., PEG) and an ionic block is mixed with an oppositely charged polyelectrolyte. Such systems have been largely developed for the loading and delivery of proteins, which can present a polyelectrolyte behavior in given pH conditions depending on their isoelectric point pI (cationic for pH < pI, anionic for pH > pI) (Figure 1, Table 1) [25]. The Kataoka group prepared micelles using a cationic poly[N-{N′-(2-aminoethyl)-2-aminoethyl}aspartamide]-PEG (pAsp(DET)-PEG) block copolymer and cytochrome C (Cyt C) protein made anionic by modifying its numerous lysine amines with citraconic anhydride (leading to carboxylate groups). PIC micelles were obtained at neutral pH with a core formed following electrostatic interactions between cationic pAsp (DET) and anionic protein, thus protecting them from degradation (Figure 1a). Interestingly, upon acidification in the endosomes, acid-triggered hydrolysis of the citraconic amides regenerated the initial protein with its strong cationic character, enabling its release from the PIC in the cytoplasm upon electrostatic repulsion [47]. This intracellular delivery was helped by the endosome-destabilization activity of the pAsp(DET) [48]. The same approach was used to develop PIC micelles based on a poly(L-lysine)-PEG block copolymer and myoglobin protein [49]. Poly(L-lysine) was made anionic through the reaction of its amines (45%) with carboxydimethyl-maleic anhydride (CDM), generating carboxylate groups, and PIC micelles were thus formed through electrostatic interactions with myoglobin (Figure 1b). The micelles were stable at pH 7.4, and micelle dissociation and protein release occurred at pH 6.5 (70% released in 24 h, against less than 20% at pH 7.4), due to the acid-mediated cleavage of the CDM amides. The PIC micelles also showed improved half-life in the blood.

PIC micelles were more recently designed as a block copolymer made by RAFT based on a PEG-block and a block bearing pendant carboxylate groups complexed with a cationic protein (lysozyme) (Figure 1b) [50]. Interestingly, the authors showed that the introduction of alkyl spacers between the vinyl backbone and the carboxylate groups improved the micelle stability in physiological conditions, but also provoked an increase in pKa of carboxylates (up to 6.3) and thus an enhanced protein release upon acidification in endosomes (pH~6), due to protonation of the carboxylates.

**Figure 2:**
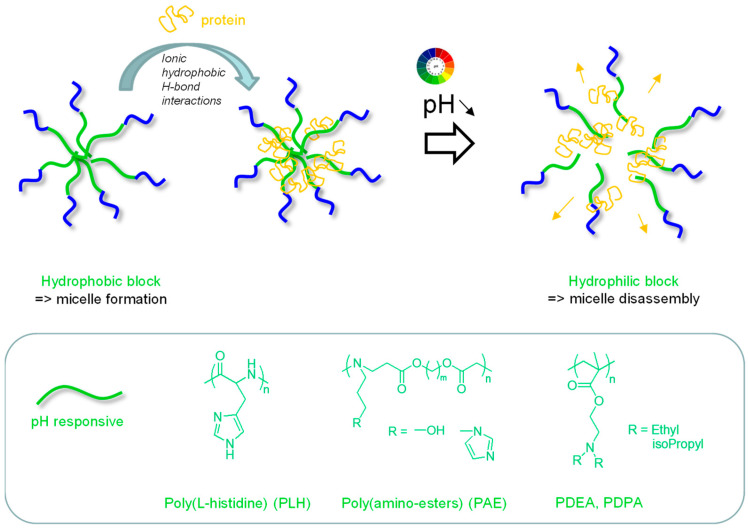
pH-responsive micelles based on block copolymers with a pH-responsive block (the box presents the typical pH responsive segments used).

pH-responsive micelles used for the physical loading of proteins can also be typically based on diblock copolymers composed of a hydrophilic block (e.g., PEG) and a block with pH-dependent properties (hydrophobic/hydrophilic) which will generally induce micelle disassembly and endosomal escape capacity (Figure 2, Table 1). Poly(L-histidine) (PLH) has been a typical example of a reported pH-responsive polymer block [51]. Hydrophobic at physiological pH 7.4, it becomes protonated and thus hydrophilic at the relevant pH range (from 7.4 to 5) thanks to a rather low pKa (~6.8), promoting micelle disassembly. In addition, it has interesting endosomolytic properties through the proton sponge effect thanks to its buffering capacity [51,52].

**Table 1:** pH-responsive micelles for protein/peptide delivery through non-covalent approach: PIC (Figure 1) and standard micelles with the pH-responsive block (Figure 2).

Copolymer	Protein/Peptide (pI)	Interactions	Results	Ref.
**PIC micelles**				
pAsp(DET)-PEG	Cytochrome C ^a^ (3.7)	electrostatic	Improved cytoplasm delivery	[47]
Poly(L-lysine) ^a^-PEG	Myoglobin (7)	electrostatic	Improved half-life in blood	[49]
Polycarboxylate-PEG	Lysozyme (11)	electrostatic	Improved release upon cell uptake	[50]
**Standard micelles**				
Poly(β-amino ester)-PEG	BSA (5) HSA (5)	hydrophobic/H-bond/electrostatic	Improved delivery in acidic tissues (ischemic area)	[53] [54]
Poly(urethane amino sulfamethazine)	SDF-1α (9.8)	hydrophobic/H-bond/electrostatic	Enhanced neurogenesis and angiogenesis (cerebral ischemia)	[55]
phosphatidylethanolamine-PEG	E5 peptide (11.4) ^b^	electrostatic	Improved CXCR4 targeting by E5 antagonist (cancer)	[56]
Polyglutamate-PEG	BSA (5)	electrostatic	Efficient delivery in cells	[57]
Polyhistidine-polyglutamate	Granzyme B (9.6) ^c^	electrostatic	Improved anti-tumor efficiency	[58]
Poly(lactide-co-glycolide)-PEI ^a^	Antimicrobial peptide ^d^	electrostatic	Biofilm eradication	[59]

^a^ Made anionic (carboxylates) through acid labile link. ^b^ Co-delivery with hydrophobic doxorubicin drug. ^c^ Co-delivery with hydrophobic docetaxel drug. ^d^ Conjugated to photosensitizer chlorin e6 for photodynamic therapy.

D. S. Lee’s group developed poly(β-amino ester)-g-PEG (PAE-g-PEG) copolymers exhibiting pH sensitivity (Figure 2) [53]. Micelles were formed at neutral pH due to the hydrophobic character of PAE, and at a pH of below 7, protonation of the tertiary amine groups led to a fully soluble copolymer and thus micelle disassembly, releasing albumin protein. The team optimized this copolymer by introducing imidazole groups in the PAE segment for improving protein association through hydrogen bonding and hydrophobic interactions and applied this carrier in a cerebral ischemia model known to exhibit an acidic environment. The rats intravenously injected with micelles loaded with Cy5.5 fluorophore-labeled albumin showed improved fluorescence in the ischemic area compared to those injected with free fluorophore-labeled albumin [54] due to micelle disassembly in an acidic environment. To note, the secondary structure of the protein was not altered by pH, as shown by circular dichroism. A similar copolymer with additional sulfamethazine units was also developed, again in a stroke context [55]. At the physiological pH of 7.4, the copolymer formed micelles encapsulating positively charged protein (SDF-1α, Stromal cell-derived factor-1α) by ionic interactions with negatively charged sulfamethazine groups. When pH decreased, ionization of the tertiary amines of the polymer led to micelle disassembly and effective release of the protein. Again, increased amounts of protein (SDF-1α) were found in the ischemic region when delivered with this copolymer carrier. Furthermore, the delivered SDF-1α enhanced neurogenesis and angiogenesis [55].

More classical amphiphilic compounds, such as phosphatidylethanolamine-PEG (PE-PEG) [60,61], have been also used to promote the pH-triggered release of proteins. Micelles from this polymer were loaded with E5 peptide, an antagonist of the CXCR4 receptor for preventing tumor metastasis [56]. The protein was encapsulated through electrostatic interactions between a slightly negatively charged polymer (phosphate groups) and a positively charged E5 peptide (isoelectric point (pI ) = 11.4) as well as hydrophobic interactions. A faster protein release was observed at pH 5 compared to pH 7.4 due to electrostatic repulsion occurring between the protein and polymer (pI = 5.9), which turned positively charged at pH 5. Typically, 80% of E5 was released in 24 h at pH 5, compared to 30% at pH 7.4. Interestingly, doxorubicin (Dox, pI: 9.06) could be co-loaded with the protein in the micelles and also released in a pH-sensitive manner. The micelles improved the targeting efficiency for CXCR4 and the efficiency of the Dox drug.

pH-sensitive micelles based on amphiphilic poly(ethylene glycol)-b-poly(L-glutamate-g-tyramine) (PEG-b-P(GA-g-Tyr)) were also developed [57]. A PEG-b-PGA copolymer was first synthesized through ring-opening polymerization (ROP) of γ-benzyl-L-glutamate-and further deprotection of the benzyl groups for subsequent grafting of tyramine on some carboxylic acids of the polyglutamic acid (PGA) block in the presence of coupling agents (EDC/NHS). Then, the pendant tyramine groups were crosslinked by an HRP-catalyzed oxidation reaction in a very dilute hydrogen peroxide solution to form core-crosslinked nanogels. BSA protein could be efficiently loaded in such a system at pH 7.4, which was surprising at first sight, as PGA and BSA are both overall negatively charged (pKa and pI of about 4.5–5). The authors explained this phenomenon by the presence of patches of positive charges on BSA even above its pI (as reported by the Ballauff group back in 2003 [62]), allowing electrostatic interactions with the negatively charged carboxyl groups of PGA. The protein was released in the pH-dependent manner, in higher amounts at pH 6.8 compared to pH 7.4, which was attributed to the protonation of the carboxyl groups of PGA, weakening the electrostatic interactions with positive patches of the protein.

### 2.2. pH-Repsonsive Micelles in Biomaterials

Increasing research works are being devoted to micelle embedding in biomaterials to improve local treatments regarding controlled release. pH-responsive micelles based on a poly (γ-glutamic acid)-b-poly(L-histidine) (PGA-PLH) copolymer loaded with both docetaxel and Granzyme B (GrB) protein as anticancer drugs were incorporated into a thermosensitive PEG-based hydrogel [58]. The cationic protein (pI~9.6) was loaded in the micelles at neutral pH through electrostatic interactions with the negatively charged PGA corona, while docetaxel was encapsulated in the neutral, hydrophobic PLH core of the micelles (Figure 2). The system, gelling in situ at body temperature, was injected peritumorally and degraded by proteinase, releasing the micelles that accumulated deeply in tumor cells. Upon acidification in the endosomes, protonation of the imidazole amine groups of the PLH block (pKa~6.8) provoked the disassembly of the micelles and endolysosomal escape (via the proton sponge effect) to release both drugs, showing a synergistic antitumor efficacy [58].

More recently, Lei et al. embedded pH-sensitive micelles in a microneedle patch made of hyaluronic acid to release an antimicrobial cationic peptide (conjugated to a photosensitizer chorine e6) preferentially in the acidic environment of a bacterial biofilm [59]. The micelles were based on poly(lactide-co-glycolide)-polyethyleneimine (PLGA-PEI), whose primary amines were functionalized with 2,3-dimaleic anhydride (DA) through amidation. This provided negatively charged micelles at the surface (due to a carboxylate group arising from DA) which became positively charged in the acidic environment due to cleavage of the pH-labile amide group from DA, thereby releasing the loaded peptide. This system showed excellent antimicrobial activity on biofilm-infected diabetic mice. Interestingly, a similar approach of negative-to-positive charge reversal induced in mildly acidic conditions (with maleic anhydride derivative) was used to recover the cationic character of antimicrobial poly(L-ornithine) at the micelle surface and thus cytotoxic efficiency selectively on tumor cells [63].

### 2.3. Thermo-Repsonsive Micelles

Thermo-responsive micelles for protein delivery have gained interest over the last few years. For example, radical copolymerization of 1-butyl-3-vinylimidazolium bromide and NIPAAm gave copolymers presenting an LCST (38.2 °C) higher than that of pure PNIPAAm (32 °C). Anionic BSA could be loaded in the copolymer micelles through electrostatic interactions with cationic imidazolium groups at body temperature and released (73% of the entrapped amount) in the temperature range of 38–42 °C (near LCST), following size contraction. This offers a nice perspective on the delivery of proteins in a hyperthermic treatment context [64].

Thermal stimulus is also interesting in developing injectable hydrogels from micelles [65,66]. The group of Mallapragada developed an amphiphilic pentablock copolymer consisting of poly(diethylaminoethyl methacrylate) (PDEA) segments at each extremity of triblock Pluronic F127 [67,68,69]. The micelles of the copolymer formed a gel at body temperature, as did unmodified Pluronic F127, through micelle association (while PDEA could also induce pH-sensitive behavior). For vaccination purposes, this system could be easily loaded with protein antigen (ovalbumin, OVA) by simple mixing at room temperature, and used as an injectable system forming an antigen depot in vivo with persistence at the site of injection for over 50 days. An improved immune response was observed (fivefold higher than that of antigen alone) until 6 weeks after administration. Notably, secondary and tertiary structures of the protein as well as its antigenicity were shown to be highly preserved. In addition, to circumvent the poor long-term stability of injectable hydrogels based on Pluronic F127 micelles, Lee et al. synthesized adamantane-conjugated Pluronic F127 and mixed them with cyclodextrin polymers to induce additional host-guest interactions [70]. The subcutaneously injected hydrogel remained stable for up to 30 days and allowed sustained release of the loaded protein such as insulin, whose profile could be controlled by varying the cyclodextrin content in the hydrogel.

### 2.4. Multiple Stimuli-Responsive Micelles

To improve their efficiency in delivering proteins to their target with optimal therapeutic effect, micelles can be designed to present responsiveness to multiple stimuli of interest.

Dual pH/sugar-responsive PIC micelles were for example developed by Ren et al. [71], using electrostatically driven self-assembly of both PEG-polylysine (cationic) and PEG-polyglutamic acid (anionic) copolymers at neutral pH. Notably, the polyglutamic acid and polylysine blocks presented phenylboronic acid and catechol groups, respectively, to induce additional core-crosslinking through boronic acid-catechol interactions, and thus improved stability and avoided the premature release of loaded protein. The Cytochrome C protein (cationic) was loaded through electrostatic interactions with the polyanion block and could be burst released in response to the endosomal acidic pH, as well as the excess of fructose sugar, due to its binding affinity with boronic groups, breaking the boronate ester crosslinks.

Dual pH/enzyme-responsive micelles such as a PEG-b-poly(2-(diisopropylamino)ethyl-methacrylate (PDPA) copolymer have recently been described for tumor vaccine purposes (Figure 2) [72]. The PDPA block contained an imidazoquinoline adjuvant as pendant groups, coupled to the backbone via a peptidic linker (GFLG) sensitive to the cathepsin B enzyme. Micelles encapsulating the ovalbumin (OVA) peptide antigen (SIINFEKL sequence) were formed. After phagocytosis of the micelles by APCs (particularly dendritic cells, DCs), the acidic environment of the endosomes led to micelle disassembly following protonation of the amines of the PDPA block, thereby releasing the antigen. Degradation of the GFLG linker by the cathepsin B present in the endosomes induced the release of imidazoquinoline, which could efficiently interact with its TLR7/8 receptor (in the inner membrane of the endosomes), and thus stimulate/maturate the DCs. This resulted in an improved cellular immune response against B16-OVA in tumor-bearing mice.

Triple pH/temperature/reduction-sensitive nanogels were developed by Li et al. using an original micelle-mediated strategy [73]. First, an amphiphilic conjugate consisting of an anionic phospholipid linked with thermoresponsive PNIPAAm was synthesized and self-assembled into micelles above the lower critical solution temperature (LCST ~ 32 °C) of PNIPAAm. The hydrophobic PNIPAAm and distearoyl groups of the phospholipid formed the core, while its phosphate groups composed the corona. Chelation of the phosphate with Ca^2+^ ions induced the formation of nanoclusters through micelle bridging. These nanoclusters were further core-crosslinked using a reduction-sensitive cysteamine (H_2_N–(CH_2_)_2_–S–S–(CH_2_)_2_–NH_2_) by reaction of its amines with the N-succinimidyl-activated esters present along the PNIPAAm chains, and calcium ions were further removed by dialysis against EDTA. An anionic nanogel was thus obtained, and efficiently loaded at neutral pH with proteins presenting with a cationic character (pI > 8, e.g., lysozyme, Cytochrome C, RNAse A). The authors showed a temperature-dependent release of RNAse A protein. However, the pH- and reduction-responsiveness of this nanogel was only studied for the release of small drugs (doxorubicin).

## 3. Covalent Association to Micelles

**Figure 3:**
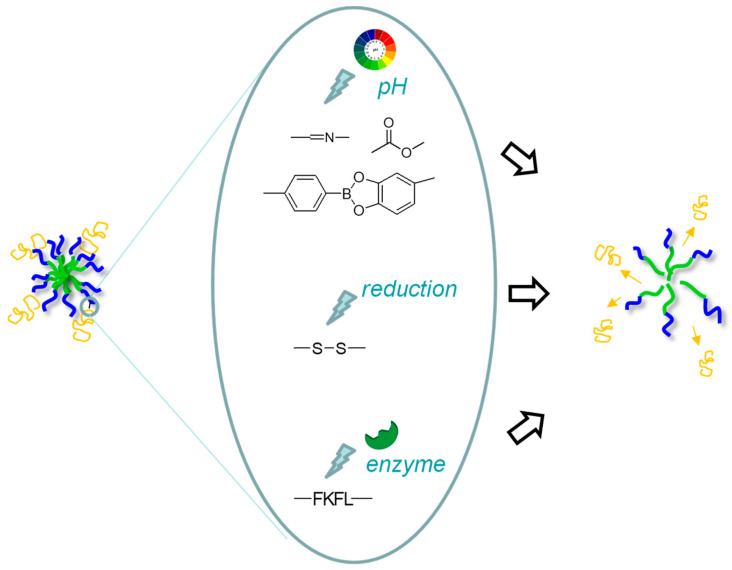
Micelles developed with stimuli (pH, reduction, enzyme)-responsive linkages to trigger protein delivery.

Covalent immobilization of proteins/peptides on micelles generally has the advantage of better stability over the non-covalent approach, avoiding premature release. Many micelles have been covalently bound to proteins/peptides through “conventional”, i.e., non-reversible chemistries such as amide bond formation [13,74,75,76], Michael additions (thiol-acrylate, thiol-maleimide) [77], or azide-alkyne click chemistry [78]. Research has more recently been dedicated to the use of stimulus-cleavable covalent bindings for triggering intracellular protein release with better control. Micelles coupled to proteins/peptides through pH, reduction, and enzyme-sensitive linkages have been particularly investigated (Figure 3, Table 2).

### 3.1. pH-Sensitive Linkages

In early works, Liu et al. have developed an aldehyde-terminated triblock copolymer of oligo(ethylene glycol)methyl ether methacrylate (OEGMA), 2-(dimethylamino)ethyl methacrylate (DMA), and 2-(diethylamino)-ethyl methacrylate (DEA) (namely, an Ald-POEGMA-*b*-PDMA-*b*-PDEA copolymer) which was used to form crosslinked micelles showing pH-dependent swelling due to the pH-sensitive PDEA block core. The aldehyde at the end of the hydrophilic shell block could be coupled to the amines of the model protein (lysozyme) to form imine pH-labile binding (Figure 3, top). However, the ability for the triggered release of the protein upon hydrolysis of the imine in mildly acidic conditions was not studied [79].

Yuba et al. have developed PLA-PEG-PLA block copolymers whose hydroxyl end-groups were conjugated to lisinopril dipeptide by ester formation in the presence of carbodiimide/DMAP coupling agents (Figure 3, top). Peptide release was not observed at pH 7.4 while 53% release occurred at pH 4, which was attributed to the acid-triggered hydrolysis of ester bonds. Interestingly, lisinopril physically entrapped in the micelles as a control was significantly released at pH 7.4, while it was negligible for the conjugated drug, showing the relevance of a covalent strategy for the stability of drug loading at physiological conditions [80].

Recently, Zhang et al. have designed smart micelles of a triblock copolymer conjugated to ribonuclease A (RNAse A) protein through a pH-reversible catechol-phenylboronic acid linkage (Figure 3, top) and encapsulating doxorubicin (Dox) [81]. The copolymer was composed of (i) a hydrophilic PEG block, (ii) an intermediate block functionalized with pendant catechol groups for conjugation of phenylboronic acid functionalized RNAse A, and (iii) a pH-sensitive block containing tertiary amine pendant groups forming the micelle core. Upon cell internalization, the latter block became hydrophilic under the acidic environment in the endosomes (due to protonation of the amine groups), inducing micelle disassembly and Dox release as well as endosomal escape through the proton sponge effect. The release of RNAse A also occurred through acidic pH-triggered cleavage of phenylboronic acid–catechol linkages. Such enhanced intracellular releases of both RNAse A and Dox synergistically enhanced anticancer efficacy in vitro and in vivo.

### 3.2. Reduction-Sensitive Linkages

Recent research has increasingly focused on the conjugation of proteins on polymer micelles through disulfide bonds to trigger their release in reductive intracellular environments. In vaccine delivery, this is of particular interest to promote the release of protein antigen in the cytosol of APCs, favoring its processing through the MHC I pathway and thus a CD8+ cellular immune response, which is highly desirable in many infectious diseases and cancer [82,94,95,96]. Since 2014, Stayton’s team has developed micelles from block amphiphilic copolymer-bearing pendant reactive pyridyl disulfide groups for protein-antigen conjugation through their thiol cysteine (disulfide formation, Figure 3, middle) and pH-responsive DMA units, i.e., bringing membrane disruptive properties at endosomal pH (protonation of tertiary amine units) to promote endosomal escape and intracellular delivery of the peptide/protein antigen [83].

Interestingly, this copolymer platform can be tuned to introduce additional immunostimulating molecules, namely ligands of APC (DC) receptors, to further improve immune response. For example, the OVA antigen-conjugated copolymer micelles could be electrostatically complexed with an immunostimulating molecule (anionic CpG) [84], resulting in an enhanced CD8+ T cell response. Sevimli et al. used a similar copolymer approach of a disulfide-conjugated antigen and pH-responsiveness but tuned the core block to be more hydrophobic to enhance the encapsulation of the immunostimulating imiquimod molecule (hydrophobic, ligand of TLR7 of DCs). The co-delivered antigen/imiquimod with micelles improved CD8+ T cell as well as IgG antibody responses [82]. Finally, a mannose ligand was also conjugated on such OVA antigen copolymer micelles to enhance DC targeting through the mannose receptor [85], inducing improved DC activation and a higher antitumor immunity in OVA-tumor-bearing mice compared to the free peptide antigen.

Notably, improvements have recently been achieved on the copolymer micelles of the Stayton group by conjugating a lytic peptide (melittin) on the micelles through disulfide bonds, which further enhanced endosomal escape and cytotoxic T cell response [86,87].

The pH-sensitive copolymer nanoplatform developed by the Stayton team was also exploited in the anticancer drug delivery context using disulfide conjugation of a proapoptotic peptide drug for effective intracellular delivery [88]. In addition, a targeting property could be introduced by binding an anti-CD22 antibody (labelled with streptavidin) on biotin end-groups of the hydrophilic block copolymer. Tumor growth was suppressed for the duration of treatment and prolonged survival in a human B-cell lymphoma xenograft mouse model was observed. In the same anticancer context, Morales-Cruz et al. have constructed a copolymer PEG-b-PLGA functionalized at the PLGA end-group by pyridyldisulfide moieties for conjugation of the cytochrome C (Cyt C) protein through a disulfide bond, and at the PEG end-group by folic acid for targeting properties. The copolymer micelles exhibited excellent stability under extracellular physiological conditions, whereas once in the intracellular reducing environment, Cyt C was released from the conjugate. The micelles promoted endosomal escape, and their potential for antitumor therapy was shown in a brain tumor model back in 2016 [89].

### 3.3. Enzyme-Sensitive Linkages

Enzyme as a stimulus for triggering the release of drugs is probably the most currently investigated, particularly in the cancer treatment context. Indeed, specific enzymes are highly expressed in particular tumor tissues [97]. The copolymer nanoplatform developed by Stayton’s team was tuned to introduce an enzyme-cleavable peptide sequence (FKFL) between the hydrophilic block and the anti-cancer proapoptotic peptide (Figure 3, bottom), instead of the disulfide linkage previously used [90]. This sequence was efficiently cleaved by the cathepsin B protease enzyme present in intracellular compartments (upregulated in many cancers), enhancing the quantity of peptide reaching tumor intracellular targets. The micelles successfully induced apoptosis in SKOV3 ovarian cancer cells in comparison to micelles from the control polymer containing a scrambled peptide sequence [90].

Su et al. prepared micelles from an azide-modified polyethylene glycol-block-polyaspartic acid(benzylamine) (azide-PEG-b-PAsp(Bz)) with encapsulated zinc phthalocyanine (ZnPC) photosensitizer for photodynamic therapy. The azide end-groups at the micelle surface were used to couple a PD-L1 antibody to block the PD-1/PD-L1 pathway and thus reactivate the immune CD8+ T cells to attack the tumor. Interestingly, the antibody was coupled through a matrix metalloprotease 2 (MMP2) enzyme-sensitive peptide, promoting antibody-triggered release in the tumor environment (rich in MM2). ZnPC was further released intracellularly and both drugs synergistically improved the antitumor effect through this immuno-photodynamic therapeutic approach [91]. Shi et al. designed amphiphiles composed of (i) a hydrophobic palmitic acid and (ii) a cationic cell-penetrating peptide (CPP) bound to a targeting peptide moiety through an MMP2-sensitive peptide linker. Self-assembling into fiber nanostructures with surface targeting moieties was obtained, which upon cleavage of the MMP2 in the tumor environment, turned to micelles presenting CPP at their surface to facilitate cell penetration across the cell membrane [98].

MMP enzymes are also upregulated in inflammatory environments, for example in the heart following myocardial infarction. Nguyen et al. developed, in this context, peptide-polymer amphiphiles based on a polynorbornene backbone and MMP-cleavable hydrophilic peptide brushes, forming micelles of about 20 nm [92]. After intravenous injection in rats, the micelles entered the infarct tissue, in which upregulation of MMP induced peptide cleavage and transition into an aggregated-like network, allowing specific accumulation in this tissue for drug delivery. The authors have very recently greatly improved the biodegradability of these amphiphiles by introducing degradable phosphoramidate segments [93].

## 4. Conclusions and Outlooks

Over the past decade, polymeric micelles have clearly appeared as attractive and relevant carriers to trigger the precise delivery of proteins/peptides to the targeted site, either through non-covalent or covalent approaches. This is largely attributed to the high flexibility in design of the copolymers, which can be tuned to present controllable charge states as well as suitable stimuli-responsive functionalities for transiently immobilizing proteins/peptides. Such success can be tempered regarding some aspects, within the context of this review: to date, PEG is still mainly used as a hydrophilic segment, which raises increasing concerns of hypersensitivity and accelerated blood clearance [99,100,101]. Another limitation is that the smart self-assembly is strongly dependent on the protein properties (e.g., pI). However, it is important to point out that this field is rapidly evolving. Indeed, many pioneer works were performed with model proteins (e.g., BSA, or ovalbumin antigen in the case of vaccine delivery) to establish the proof of concept of the smart releasing system, mainly exemplified by in vitro studies. Nowadays, more relevant and diverse proteins of interest requiring a delivery system are currently envisioned with ad hoc and well-defined copolymer micelle design, and investigated through deeper in vivo studies, as highlighted in this review. The convincing biological performance obtained in many pathologic contexts (e.g., vaccines, cancer, inflammation) will foster works closer to the clinical application. Undoubtedly, these versatile nano-systems, with their inherent ease of preparation, scalability, and high reproducibility (moreover improvable with the current inputs of microfluidics techniques) will play a major role in the development of the next generation of protein therapeutics.

## Figures and Tables

**Table 2:** pH-, reduction-, and enzyme-responsive micelles for protein/peptide delivery through the covalent approach (see Figure 3).

Copolymer	Protein/Peptide	Stimulus	Linkage	Results	Ref.
POEGMA-PDMA-PDEA	Lysozyme	pH	Imine	Bioconjugation assessment	[79]
PLA-PEG-PLA	Lisinopril	pH	Ester	Improved drug loading stability at pH 7.4 (and release at pH 4)	[80]
Polyglutamate-PEG based	Ribonuclease A ^a^	pH	phenylboronic acid–catechol	Enhanced intracellular release, enhanced anticancer efficacy	[81]
PDPA/PDMA based	Ovalbumin Lytic peptide	Reduction	Disulfide	Improved immune responses (vaccines)	[82,83,84,85,86,87]
	Proapoptotic peptide (BIM)	Reduction	Disulfide	Suppression of tumor growth	[88]
Poly(lactide-co-glycolide)-PEG	Cytochrome C	Reduction	Disulfide	Endosomal escape, potential for antitumor therapy (brain model)	[89]
PDMA/PDPA based	Proapoptotic peptide (BIM)	Enzyme (cathepsin B)	FKFL cleavable sequence	Improved intracellular delivery, Successful cancer cell apoptosis	[90]
PAsp(Bz)-PEG	PD-L1 antibody ^b^	Enzyme (MMP2)	GGPLGVRGG cleavable sequence	Improved intracellular delivery, improved antitumor effect	[91]
Polynorbornene based	-	Enzyme (MMP2/9)	GPLGLAG cleavable sequence	Specific accumulation in infarcted heart	[92,93]

^a^ Co-delivery with hydrophobic doxorubicin drug. ^b^ Co-delivery with hydrophobic Zn phthalocyanine photosensitizer.
